# ENLIGHT: A consensus checklist for reporting laboratory-based studies on the non-visual effects of light in humans

**DOI:** 10.1016/j.ebiom.2023.104889

**Published:** 2023-12-02

**Authors:** Manuel Spitschan, Laura Kervezee, Renske Lok, Elise McGlashan, Raymond P. Najjar, ENLIGHT Consortium, Annette E. Allen, Marilyne Andersen, Salvador Bará, Peter Blattner, Christine Blume, Diane B. Boivin, María-Ángeles Bonmatí-Carrión, Kai Broszio, Timothy M. Brown, Sarah Laxhmi Chellappa, Jeanne F. Duffy, Taisuke Eto, Erin Flynn-Evans, Steve Fotios, Virginie Gabel, Corrado Garbazza, Gena Glickman, Marijke C. Gordijn, John P. Hanifin, Lauren Hartstein, Michael Herf, Shigekazu Higuchi, Cassie J. Hilditch, Kevin W. Houser, Anya Hurlbert, Monique K. LeBourgeois, Steven Lockley, Robert Lucas, Claudia R.C. Moreno, Mirjam Münch, Ludovic S. Mure, Stuart Peirson, Shadab Rahman, Victoria L. Revell, Roberto G. Rodriguez, Kathryn Roecklein, A.V. Rukmini, John Sammarco, Nayantara Santhi, Luc J.M. Schlangen, Isabel Schöllhorn, Katherine M. Sharkey, Debra J. Skene, Tracey L. Sletten, Karin C.H.J. Smolders, Oliver Stefani, Julia E. Stone, Petteri Teikari, Michael Terman, Khanh Tran Quoc, Kazuo Tsubota, Ljiljana Udovicic, Gilles Vandewalle, Jennifer A. Veitch, CélineVetter, Lisa M. Wu, Johannes Zauner, Jamie Zeitzer

**Affiliations:** a TUM School of Medicine & Health, Department of Health and Sport Sciences, Technical University of Munich, Munich, Germany; b TUM Institute for Advanced Study (TUM-IAS), Technical University of Munich, Garching, Germany; c Max Planck Institute for Biological Cybernetics, Max Planck Research Group Translational Sensory & Circadian Neuroscience, Tübingen, Germany; d TUMCREATE, Singapore, Singapore; e Laboratory for Neurophysiology, Department of Cellular and Chemical Biology, Leiden University Medical Center, Leiden, Netherlands; f Department of Psychiatry and Behavioral Sciences, Stanford University, Stanford, USA; g Melbourne School of Psychological Sciences, The University of Melbourne, Parkville, Victoria, Australia; h School of Psychological Science and Turner Institute for Brain and Mental Health, Monash University, Melbourne, Australia; i Department of Ophthalmology and Department of Biomedical Engineering, National University of Singapore, Singapore, Singapore; j Center for Innovation & Precision Eye Health, National University of Singapore, Singapore, Singapore; k Singapore Eye Research Institute, Singapore, Singapore; l Ophthalmology and Visual Sciences Academic Clinical Programme, Duke-NUS Medical School, Singapore, Singapore

**Keywords:** Reporting guidelines, Non-visual effects of light, Circadian rhythms, Sleep, Interventions, Light

## Abstract

**Background:**

There is no consensus on reporting light characteristics in studies investigating non-visual responses to light. This project aimed to develop a reporting checklist for laboratory-based investigations on the impact of light on non-visual physiology.

**Methods:**

A four-step modified Delphi process (three questionnaire-based feedback rounds and one face-to-face group discussion) involving international experts was conducted to reach consensus on the items to be included in the checklist. Following the consensus process, the resulting checklist was tested in a pilot phase with independent experts.

**Findings:**

An initial list of 61 items related to reporting light-based interventions was condensed to a final checklist containing 25 items, based upon consensus among experts (final n = 60). Nine items were deemed necessary to report regardless of research question or context. A description of each item is provided in the accompanying Explanation and Elaboration (E&E) document. The independent pilot testing phase led to minor textual clarifications in the checklist and E&E document.

**Interpretation:**

The ENLIGHT Checklist is the first consensus-based checklist for documenting and reporting ocular light-based interventions for human studies. The implementation of the checklist will enhance the impact of light-based research by ensuring comprehensive documentation, enhancing reproducibility, and enabling data aggregation across studies.

**Funding:**

Network of European Institutes for Advanced Study (NETIAS) Constructive Advanced Thinking (CAT) programme; Sir Henry 10.13039/100004440Wellcome Postdoctoral Fellowship (Wellcome Trust, 204686/Z/16/Z); 10.13039/501100001826Netherlands Organisation for Health Research and Development VENI fellowship (2020–09150161910128); 10.13039/100000005U.S. Department of Defense Grant (W81XWH-16-1-0223); 10.13039/501100001352National University of Singapore (NUHSRO/2022/038/Startup/08); and 10.13039/501100001381National Research Foundation Singapore (NRF2022-THE004-0002).

**Research in context**

**Evidence before this study**

Prior to this study, there was a lack of consensus on how to report light characteristics in studies investigating non-visual responses to light. This absence of standardized reporting hindered the comparability and reproducibility of research findings in the field of light-based interventions for human studies.

**Added value of this study**

This study's primary contribution lies in the development of the ENLIGHT Checklist, a consensus-based framework for documenting and reporting ocular light-based interventions in laboratory-based research. The creation of these guidelines addresses the existing gap in standardized reporting and enhances the quality and utility of research by ensuring comprehensive documentation, reproducibility, and facilitating data aggregation across studies.

**Implications of all the available evidence**

The establishment of the ENLIGHT Checklist is expected to have far-reaching implications in studies employing ocular light exposure interventions. These guidelines will promote greater consistency and transparency in reporting, making it easier for researchers to build upon existing knowledge and advance our understanding of the non-visual effects of light on human physiology and well-being. Furthermore, the potential for improved data aggregation across studies will enable researchers and policymakers to make more informed decisions about the use of light interventions in various contexts, such as healthcare, architecture, and environmental design.

## Introduction

Light exerts powerful effects on our physiology and behavior beyond enabling vision.^1^^,^^2^ One of the primary non-visual functions of light is the synchronization of the circadian clock.^3^ In addition, light exposure influences alertness,^4^^,^^5^ mood,^2^ and cognitive brain function.^6^ The impact of light on these non-visual functions depends on the intensity, timing, temporal pattern as well as spectral properties of light exposure. Furthermore, the non-visual effects of light vary significantly among individuals.^7^, ^8^, ^9^ Therefore, even minor differences in the intensity, pattern, spectral quality, or timing of light stimuli in clinical and basic research studies may result in substantial differences in the response observed, signifying the need for standardized measurement and reporting practices in lighting research.

Many metrics are available to quantify light exposure in intervention studies on the non-visual effects of light. These include (ir)radiance (in energy or photon units) (il)luminance, luminous flux, melanopic quantities, chromaticity, correlated color temperature, and the tabulated spectral power distribution. The large diversity of available metrics inherently leads to a high risk of inconsistent reporting across studies. Indeed, a careful review of a selection of 19 articles referenced in a recent publication on the non-visual effects of light^10^ showed that while all articles reported at least one measure of light intensity (either irradiance, radiance, illuminance, luminance, or luminance flux), no single measure of light intensity was reported across articles. In addition, none of the other metrics were reported by all 19 articles (see Supplemental Table S1 for details). Inconsistent reporting reduces reproducibility and complicates direct comparisons between studies, precluding the conduct of meta-analyses and further evidence synthesis in the field. As neurobiological studies on the non-visual effects of light are highly resource-intensive, often taking multiple years to complete, improved reporting would represent a significant step forward for the field.

The development of reporting checklists via consensus processes involving large groups of experts in the field represents an established method to improve reporting in biomedical research.^11^ While several reporting schemes have been proposed for studies on the non-visual effects of light,^12^, ^13^, ^14^, ^15^ some dating as far back as 1991,^14^ none of them were based on consensus among experts. Motivated by the inconsistencies in reporting in the field and the lack of consensus-derived reporting checklist, our aim was to produce a specific reporting checklist and accompanying Elaboration and Explanation (E&E) document for light and study characteristics in laboratory-based interventions studying the effects of ocular light exposure on non-visual physiology in human research participants. In pursuit of this objective, we adopted a rigorous and systematic approach, employing a four-step modified Delphi method^16^ that involved engaging a panel of experts in non-visual effects of light field. The ENLIGHT (**E**xpert **N**etwork on **LIGHT** Interventions: **ENLIGHT**) Checklist is intended to provide guidance to authors and to assist reviewers, editors, and readers in appraising the completeness and applicability of study findings, as well as enable the synthesis of data across published work.

## Methods

### Ethical approval and registration

The ENLIGHT project received ethical approval from the University of Oxford Medical Sciences Interdivisional Research Ethics Committee (MS IDREC) (approval number R78618/RE001). All participants gave informed consent to be part of this study. The study was registered with the EQUATOR Network (https://www.equator-network.org/library/reporting-guidelines-under-development/reporting-guidelines-under-development-for-other-study-designs/#ENLIGHT). The protocol was pre-registered on the Open Science Framework (https://doi.org/10.17605/OSF.IO/XR965). There were no deviations from the pre-registered protocol. The thinking-aloud sessions and written feedback, which took place after provisional finalization of the checklist, were not part of the pre-registration.

### Code, materials and data availability

All code, materials and data are available on GitHub (https://github.com/ENLIGHT-Project/), containing the survey configurations used in JISC Online Surveys and PDF printouts (https://github.com/ENLIGHT-Project/ENLIGHT-Survey), the data (https://github.com/ENLIGHT-Project/ENLIGHT-Data), and the archival checklist and E&E document (https://github.com/ENLIGHT-Project/ENLIGHT-Checklist). All data and materials are licensed under CC-BY-NC-ND, and all code is licensed under GNU General Public License v3.0 (GPLv3). The project website can be found at https://enlight-statement.org/.

### Protocol and ENLIGHT steering committee

The ENLIGHT Checklist and E&E document were developed in accordance with the EQUATOR toolkit for developing a reporting guideline (https://www.equator-network.org/) through a modified Delphi consensus process that took place between December 2021 and December 2022 consisting of (1) a pre-round where the project team went through a literature review, identified a set of potential reporting-related items and a group of participants with an established track record in light-based interventions and (2) four feedback rounds (three questionnaire-based and one face to face discussion) detailed in the procedure section below.

A five-member steering committee was established to coordinate the development process of ENLIGHT, consisting of scientists with expertise in the visual and non-visual effects of light. The ENLIGHT Steering Committee consists of M.S., a visual neuroscientist with expertise in visual and circadian neuroscience, L.K., a chronobiologist with expertise in human physiology and neuroscience, R.L., a chronobiologist with expertise in light, circadian rhythms, and alertness, E.M., a chronobiologist with expertise in light, circadian rhythms, and mental health, and R.P.N., a visual neuroscientist with expertise in circadian biology, light, and ocular diseases. The ENLIGHT Steering Committee coordinated the modified Delphi consensus process, including selecting participants, designing and distributing the online surveys for the modified Delphi consensus process, organizing and moderating the face-to-face consensus meetings, analyzing data, and drafting the ENLIGHT Checklist and complementary E&E document. The ENLIGHT Steering Committee had regular online meetings and met in person in three one-week visits to coordinate and finalize the Delphi process as well as the ENLIGHT Checklist and E&E document.

### Participants and consortium formation

Potential participants with experience in laboratory-based studies in human participants on the non-visual effects of light were invited to participate in this exercise through purposive sampling. To ensure the broadest representation of feedback, a concerted effort was made to invite participants at different career stages, of any sex or gender, from different geographical locations, and working at a variety of academic and industrial institutions. In the invitation email, participants were also asked to provide recommendations and contact details of additional suitable participants to be included in the exercise. Participants who completed Rounds 1, 2, and 4 of the consensus process were invited to join the ENLIGHT Consortium and those who opted to join are acknowledged by name within a group authorship model (Supplemental Table S2). Participants were not remunerated for their participation.

### Procedure

#### Preliminary work: selection of concepts

We identified concepts and items that could be used in the ENLIGHT Checklist. These were based on the steering committee's domain knowledge, as well as by consulting the relevant literature. In identifying concepts, we consulted a number of key references. As early as 1991, Remé, Menozzi and Krueger^14^ proposed a reporting scheme. Spitschan et al.^13^ proposed a series of items for reporting interventions involving light in the field of chronobiology, sleep research, and environmental psychology. Knoop et al.^15^ developed a workflow for identifying which quantities to measure and report in research on the non-visual effects of light. Finally, the International Commission on Illumination (CIE) released a technical note, CIE TN 011:2020, discussing items to report in studies on ipRGC-influenced responses to light.^12^

Considering both specific metrics and study protocol aspects identified by these resources, as well as broad aspects of study design or lighting, which are consistent across the existing resources, an initial pool of 61 items was generated. These items covered protocol, experimental, measurement, instrument, and source level characteristics, as well as the spectral, photometric, color, spatial and temporal aspects of the light source.

#### Round 1: importance of preliminary items, gathering of additional items, and initial draft of checklist

In Round 1, participants were invited by email to complete an online survey using a web-based survey tool hosted by the University of Oxford (JISC Online Surveys). After reading a participant information sheet, participants were asked to complete an informed consent form and then to rate the importance of a set of preliminary items identified in the *Pre-round* on a 1–7 scale, with the following options: 1—Very unimportant, 2—Quite unimportant, 3—Unimportant, 4—Neither unimportant nor important, 5—Important, 6—Quite important, 7—Very important. To allow for participants being unable to assess the importance of a specific item, we also included a further open category (X—Don't know or recognize this quantity and cannot evaluate).

Additionally, participants were asked to identify any items that were missing from the initial list of items, which they deemed to be important. The goal of this round was to obtain quantitative insights into the importance of specific concepts and to gather additional items that should have been included in the preliminary work. The threshold for definite inclusion in Round 2 was that ≥75% of all responses (including those that responded “X—Don't know or recognize this quantity and cannot evaluate”) needed to be above the midpoint of the scale (i.e., >4). Items with ≥75% of responses below the midpoint (<4) were excluded. Items which did not fall into these threshold-based categories were reserved for further evaluation in Round 2 and face-to-face discussions in Round 3. The consensus threshold of 75% was defined in our pre-registered protocol and was based on the median threshold that is used in other consensus-based projects.^17^ At the conclusion of Round 1, an initial draft checklist was made based on the ratings from experts for items within the predefined categories.

In Round 1, we also probed the demographic characteristics of participants, and asked about their sex (“What is your sex?”, with options “Male”, “Female” and “Prefer not to say”) and gender (“Is the gender you identify with the same as your sex registered at birth?”, with options “Yes”, “No” and “Prefer not to say”, their holding of a PhD (yes/no) and year of PhD award, their main role (“What is your main role?”, with options “Principal Investigator”, “Postdoc”, “Doctoral student”, “Master's student”, “Research assistant” and “Other”), the model organism they work with (“Human”, “Mouse” or “Other), and the country/countries they currently live and work in (free text). The exact questions can be viewed in the GitHub repository for the survey (https://github.com/ENLIGHT-Project/ENLIGHT-Survey).

#### Round 2: draft checklist evaluation and format specification

In Round 2, the draft checklist was circulated to experts for initial feedback. We asked experts to indicate whether there were any items from Round 1 which were not included in the draft that they feel should have been and whether there were any items included in the draft which should not have been. Additionally, we asked experts to indicate the preferred format (text, table figure) for each of the items included in the draft checklist. New items identified in Round 1 were introduced in Round 2, and expert consensus was sought for their inclusion. A draft checklist and results of Round 2 were then circulated to participants, along with an invitation to join the face-to-face discussions in Round 3.

#### Round 3: face-to-face feedback and discussion sessions

In Round 3, the steering committee led 1-h discussion sessions with small groups of participants via Zoom video calls. Only participants who completed both Round 1 and Round 2 were invited to participate. At least two members of the steering committee attended each session: one chairing the session and one taking notes. The sessions were also recorded so that they could be later reviewed if necessary. The sessions involved semi-structured discussions around the following themes: (1) clarifying any open questions or concerns; (2) discussing the scope of the E&E document and accompanying checklist; and (3) discussing dissemination and impact of the checklist and E&E document. Following the discussion sessions in Round 3, the checklist was revised to incorporate the feedback of the expert panel, and the accompanying E&E document was written.

#### Round 4: essential reporting items and E&E document

In Round 4, we sought consensus on which items on the checklist should be deemed essential to report regardless of context. That is, all items that were rated by more than 75% of the participants were considered essential, and the “Not applicable” option was removed for these items in the final checklist. In addition, we also requested qualitative feedback on the final checklist draft and accompanying E&E document. Lastly, we evaluated the satisfaction of our participants with the process, and the resulting checklist and E&E document. Participants who completed both Round 1 and Round 2 (but not necessarily Round 3) were invited to participate.

Following Round 4, the ENLIGHT Steering Committee prepared the provisional checklist and E&E document, which were distributed to participants who accepted the invitation to join the ENLIGHT Consortium.

#### Independent pilot testing phase

To refine the documents and add an additional level of validation, we conducted an independent pilot testing phase. For this round, we followed a two-pronged approach, involving experts that were not part of the consensus process.

*Written feedback:* We invited a group of experts of diverse experience levels (PI, post-doc, PhD student) to read through and give written feedback on the ENLIGHT Checklist and E&E document through an Excel spreadsheet answering the following questions:

• Is the layout of the ENLIGHT Checklist clear and easy to navigate?
• Is the layout of the ENLIGHT E&E document clear and easy to navigate?
• Are there any improvements or modifications you would suggest to enhance its usability and make it more comprehensible?
• Does the terminology used in the ENLIGHT Checklist and E&E document accurately convey the intended meaning and instructions?
• Are there any terms or phrases in the ENLIGHT E&E document that you find unclear or ambiguous that could be expanded?

*Thinking-aloud sessions:* To probe the usability of the documents, we invited an additional group of experts who had recently published or completed empirical work, using light-based interventions to-participate in 30-min semi-structured thinking-aloud sessions via Zoom. In these sessions, participants of diverse experience levels (Principal investigator, post-doc, PhD student) gave feedback on their experience on completing the checklist for their respective study. Participants were given the following questions in advance:

• Were you able to complete the checklist?
• How long did it take you to do this (in minutes)?
• On a scale of 1–5 (1: poor, 2: fair, 3: good, 4: very good, 5: excellent), how would you rate your experience in completing the checklist?
• What did you like about it?
• What did you dislike about it?
• Did the E&E document effectively clarify the items within the Checklist?
• Do you think that the Checklist and E&E document will improve reporting in the field?
• What year did you receive your (first) PhD or other research-based doctoral degree (if you have one)?

These questions were then discussed in a semi-structured fashion. Sessions took place via Zoom with two Steering Committee members. Notes were taken, which were then later analysed.

#### Statistics

All statistics were descriptive and were implemented in R (version 4.2.2).

#### Finalization

Following the independent pilot testing phase, the ENLIGHT Steering Committee finalized the checklist and E&E document through synchronous discussions.

#### Role of funders

Funding sources had no role in the study design; in the collection, analysis, and interpretation of data; in the writing of the report; or in the decision to submit the paper for publication.

## Results

### Novel consensus checklist for light-based interventions

The ENLIGHT Checklist was developed using a four-step modified Delphi process.^16^ This consisted of preliminary work to identify an appropriate list of initial items for evaluation (see **Methods** and Supplementary Table S2), three survey-based rounds (Round 1, 2 and 4), and one round of face-to-face discussion (Round 3). The goal of Round 1 was to assess ratings of the importance of the list of items identified in the preliminary work and the initial drafting of the checklist. In Round 2, experts were asked to evaluate the initial draft of the checklist, and to provide input on the preferred format of items. Round 3 consisted of face-to-face discussions with experts to clarify questions or concerns from participants, discuss the scope of the E&E document accompanying the checklist, and discuss how to maximize the impact and adoption of the ENLIGHT Checklist. In Round 4, the final version of the checklist and accompanying E&E document were reviewed, and experts were asked to indicate which items should be mandatory. Resulting from this round, a provisionally finalized checklist and E&E document was subjected to an independent pilot validation round, leading to final corrections and adjustments of the documents. The final ENLIGHT Checklist is a convenient, fillable form-based PDF, accompanied by a detailed E&E document.

### Round 1: importance of preliminary items, gathering of additional items, and initial checklist drafting

**Fig. 1:**
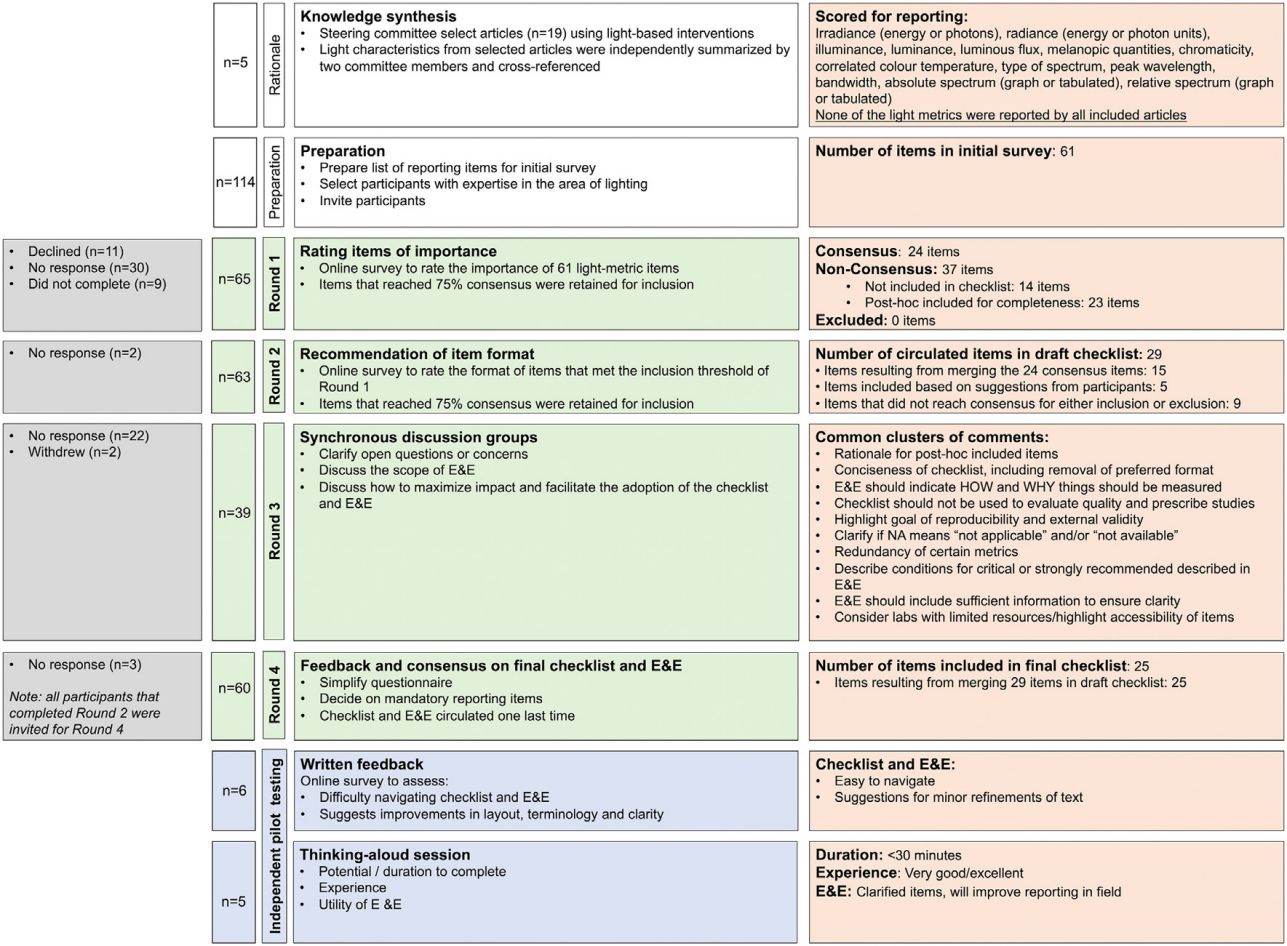
General overview of participant inclusion, the consensus rounds, and item selection for the checklist.

Of the 115 invited experts, 65 participants completed the first survey (see Fig. 1 for a flow chart of participant recruitment and Table 1 for demographic information).

**Table 1:** Demographic characteristics of participants of Round 1 (n = 65).

Included experts—Round 1 (n = 65)	
*Sex, n (%)*	
Female	36 (55.4%)
Male	28 (43.1%)
Prefer not to say	1 (1.5%)
*Current continent, n (%)*	
Europe	33 (50.8%)
North America	23 (35.4%)
Asia	5 (7.7%)
Australia	2 (3.1%)
South America	2 (3.1%)
*PhD degree, n (%)*	
Yes	61 (93.8%)
No	4 (6.2%)
*Position, n (%)*	
Principal Investigator	49 (75.4%)
Postdoc	10 (15.4%)
Doctoral student	3 (4.6%)
Other	3 (4.6%)
*Model organism, n (%)*	
Human	63 (96.9%)
Non-human only	2 (3.1%)
*Self-rated knowledge of measuring light interventions, n (%)*	
High	24 (36.9%)
Above average	23 (35.4%)
Average	16 (24.6%)
Poor	2 (3.1%)
Very poor	0 (0%)
*Self-rated knowledge of documenting light interventions, n (%)*	
High	20 (30.8%)
Above average	22 (33.8%)
Average	20 (30.8%)
Poor	3 (4.6%)
Very poor	0 (0%)

**Fig. 2:**
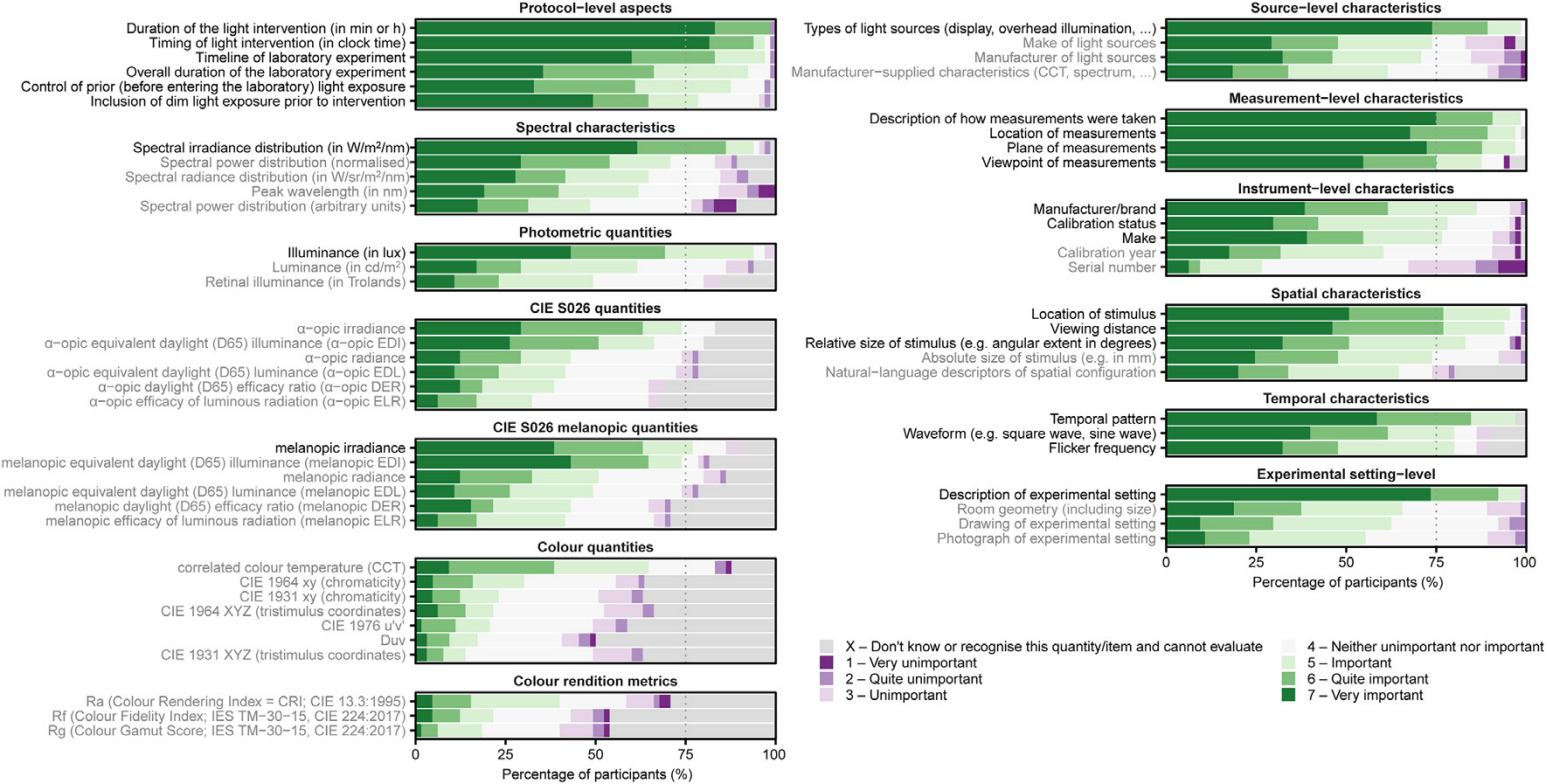
Ratings of initial list of 61 potential checklist items as unimportant–important by participants in Round 1 (n = 65 participants). Items in black: items that reached the consensus threshold for inclusion. Items in gray: items that did not reach the threshold for either inclusion or exclusion.

Participants rated the importance of 61 items and quantities for reporting on a scale from 1 (very unimportant) to 7 (very important) across 12 domains (Fig. 2). Twenty-four items reached the threshold for definite inclusion, i.e., these were rated with a score of at least “5—important” by ≥75% of participants. The 37 remaining items were rated as either “unimportant” (a score of less than 3) or “unknown”, with the exact per-item percentage ranging between 8 and 60%. None of the items were rated “unimportant” by ≥ 75% of experts and therefore, none met the threshold for definite exclusion.

In Round 1, participants also had the opportunity to suggest additional items for the checklist and provide open-ended feedback. Three clusters of common feedback were identified:

• **Suggestions for additional checklist items.** These items were mostly related to observer-level characteristics specific to light interventions (e.g., pupil dilation, ocular functioning, and timing of light interventions relative to individual circadian or sleep time).
• **Scope of checklist.** The experts noted that the importance and relevance of items highly depend on the specific study.
• **Organization of the checklist.** The experts noted that the checklist should be concise, simple to use, and not add additional burden on researchers for documenting their research.

Based on the last concern, it was decided to condense and combine some of the 24 items that reached consensus for inclusion, resulting in 15 items. Subsequently, a draft checklist was created that contained 29 items. This included the 15 condensed items that reached consensus and five new items based on suggestions from participants. After internal discussions among the ENLIGHT Steering Committee, an additional nine items that did not reach consensus for *either* inclusion or exclusion were also included in the 29-item checklist to ensure the checklist covered all major aspects of lighting, as for some aspects, no individual items reached consensus at this stage.

### Round 2: draft checklist evaluation and format specification

Sixty-three participants (97% of participants that completed Round 1) completed Round 2. None of the nine items that were additionally added to the initial draft of the checklist were rated by the majority as “should not have been included” (all percentages below 31%; Supplemental Figure S1A), and no items from Round 1, that were excluded from the initial draft, were rated as “should have been included” by the majority of experts (all percentages below 45%; Supplemental Figure S1B), suggesting that there was support for the initial draft. Consequently, all 29 items were retained for discussion in Round 3.

In addition, participants were asked to indicate their preference for the reporting format of the different items in the draft checklist. For 18 items, text was the preferred format, while a figure was preferred for 3 items and a table for 4 items (Supplemental Figure S2). For example, “figure” was the preferred format to report the timeline of the experiment, while “table” was preferred to report α-opic (ir)radiances.

### Round 3: synchronous discussions

The 63 participants who completed Round 2 were invited to participate in the synchronous discussions (Round 3 of the modified Delphi process). Participants who agreed to participate (n = 41) were split into six working groups that ranged in size from 4 to 11 participants, with a median of 7 participants per group. One video call was held for each group. Two members of the ENLIGHT Steering Committee (one moderator and one note-taker) participated in each call. Overall, 39 out of 41 participants attended their respective video calls. Two participants withdrew after being allocated to a group. The synchronous discussions focused on three objectives: (1) To clarify any open questions or concerns from participants; (2) To discuss the scope of the E&E document accompanying the checklist; and (3) To discuss how to maximize impact and facilitate the adoption of the ENLIGHT Checklist and E&E document. Two Steering Committee members reviewed video call minutes (R.L., L.K.). Recurrent comments and discussion points raised by participants were identified, discussed by the ENLIGHT Steering Committee, distilled, and grouped under ten common themes raised by multiple participants, within the three objectives of this round (Supplementary Table S3). These themes were used to simplify, condense, and improve the checklist, as well as to inform the preparation of the E&E document. Feedback on actions taken by the Steering Committee was provided to participants in Round 4 of the Delphi Process.

Following Round 3 of the Delphi process, the 29 items in the initial draft checklist were condensed to 25 items based on the feedback to further simplify and shorten the checklist. Furthermore, the wording of nine checklist items was improved. For example, “flicker frequency” was replaced by “flash frequency” to clarify that this item pertains to the intentional temporal pattern of the light stimulus. Likewise, the item ‘pupil dilation’ was reworded to “pupil size and/or dilation” to clarify that this item relates to both the description of any methods used to pharmacologically dilate the pupil as well as any methods used to measure and/or control for pupil size. In addition, based on specific comments by participants, general textual/structural improvements were made in the checklist, including (1) the removal of text/table/figure designation; (2) the specification that all light sources used should be reported; and (3) replacement of the term “light intensity” to “light level” for accuracy.

### Round 4: provisional finalization of checklist and E&E document

**Fig. 3:**
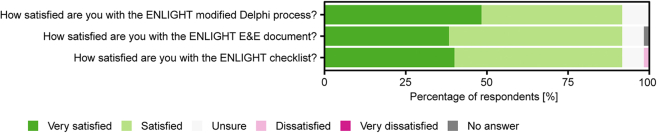
Ratings of participant satisfaction with the ENLIGHT process, E&E document, and final checklist (n = 60 participants in Round 4).

In Round 4, all participants that had completed Round 2 were asked to vote on which items on the checklist they deemed essential to be reported regardless of experimental context and to provide qualitative feedback on the final checklist and accompanying E&E document. In total, 60 participants completed this round. Nine items reached consensus on being essential, i.e., these items were rated by more than 75% of the participants as essential (Supplemental Figure S3). Based on open-ended feedback, minor textual changes were made to the checklist for accuracy. These changes included renaming the items ‘color quantities' and ‘color rendition metrics' to ‘color appearance quantities' and ‘color rendering metrics', respectively. Following feedback from the participants, some references in the accompanying E&E document were either removed or replaced with better-suiting ones. Lastly, 92% of participants reported being satisfied or very satisfied with the consensus process as well as the final checklist and E&E document (Fig. 3).

### Independent pilot testing phase

In the written feedback round, all participants (n = 6) indicated that both the checklist and E&E were clear and easy to navigate. All gave detailed feedback and suggestions for modifications of the checklist and E&E document. These comments, largely requesting clarifications or refinement of text in the E&E document, were incorporated in a revision round by the ENLIGHT Steering Committee. All respondents had a PhD and were active or retired principal investigators.

Five participants participated in the semi-structured thinking-aloud sessions. On average, participants reported completing the checklist in less than half an hour (mean±1SD 22 ± 8.37 min; range: 10 to 30 minutes) and rated their experience in completing the checklist as very good/excellent (mean±1SD 4.8 ± 0.27 on a scale from 1 to 5). All participants agreed that the E&E document effectively clarify the items within the checklist and that the Checklist and E&E document will improve reporting in the field. All participants either had their PhD (n = 4) or had just submitted their dissertation (n = 1).

### Final ENLIGHT checklist and E&E document

A list of items included in the final checklist and a short description of each item is presented in Table 2. A fillable checklist is available as Supplemental File S1. The final E&E document is available as Supplemental File S2.

**Table 2:** Items in final ENLIGHT checklist.

Item	Description
**A. Study characteristics**	
*A.1. Protocol-level characteristics*	
Description of experimental setting^a^	Describe the experimental setting (e.g., room geometry).
Timeline of experiment (including timing and duration of light)^a^	Provide an overview of the timing of key study events, including timing and duration of light exposure.
Pre-laboratory sleep-wake/rest-activity behaviour	Describe the pre-laboratory sleep-wake or rest-activity behaviour (e.g., any measurement of participants' sleep-wake or rest-activity behaviour prior to entering the laboratory).
Pre-laboratory light exposure	Describe the pre-laboratory light exposure, including whether participants were given any instructions related to light exposure.
Immediate prior light exposure (in laboratory)	Describe the in-laboratory light conditions immediately prior to the experimental light exposure.
*A.2. Measurement-level characteristics*	
Measurement plane (e.g., horizontal or vertical)^a^	Describe the plane in which light measurement(s) were performed.
Measurement viewpoint and location^a^	Describe the location and direction at which the light sensor was placed during light measurements.
Type, make and manufacturer of the measurement instrument^a^	Describe the instrument being used to take each light measurement, including the manufacturer, type, make and model of the device.
Calibration status of the instrument	Describe the calibration status of the light sensor that was used to take each light measurement.
*A.3. Participant-level characteristics*	
Ocular health and functioning^a^	Provide any details on health and functioning of the participants' eyes.
Pupil size and/or dilation	Describe any pupil size measurements and/or whether pupils were pharmacologically dilated during the experimental protocol.
Relative time (e.g., to circadian phase or sleep)	Describe the time of the experimental light exposure relative to the participants' sleep or circadian timing.
**B. Light characteristics**	
*B.1. Light sources*	
Light source type(s)^a^	Tick all relevant boxes to indicate the type(s) of background and experimental light sources used in the study.
Type, make and manufacturer of the light source^a^	Describe the type, make, and manufacturer of the light source(s) used in the study.
Use of wearable filtering apparatus (e.g., blue-blocking glasses)	Describe any wearable device(s) that modifies the absolute flux level or relative spectral distribution, or both, of light passing through it.
*B.2. Light level characteristics*	
Illuminance (lux) and/or luminance (cd/m^2^)^a^^,^^b^	Provide the illuminance and/or luminance of the experimental light condition(s) used in the study.
Spectral irradiance and/or radiance distribution^b^	Provide the spectral irradiance and/or radiance distribution of the experimental light condition(s) used in the study.
α−opic irradiance and/or radiance (including melanopic)^b^	Provide the α−opic irradiance and/or radiance of the experimental light condition(s) used in the study.
α−opic equivalent daylight illuminance and/or luminance (EDI/EDL, including melanopic)^b^	Provide the α−opic equivalent daylight illuminance and/or luminance of the experimental light condition(s) used in the study.
*B.3. Colour characteristics*	
Peak wavelength and bandwidth	Provide the peak wavelength and bandwidth of the experimental light condition(s). Note that these metrics are most relevant for monochromatic or narrowband light sources.
Colour appearance quantities (any)	Provide colour appearance quantities of the experimental light condition(s), such as any metric describing position in a chromaticity diagram or color space, or correlated colour temperature, CCT (Tc).
Colour rendering metrics (any)	Provide any colour rendering metrics, such as the Colour Fidelity Index, Rf.
*B.4. Temporal and spatial characteristics*	
Location of stimulus and viewing distance^a^	Describe the location of the light stimulus relative to the participant, and/or the relative distance between the light stimulus and the participant.
Temporal pattern (including flash frequency and waveform)	Describe the temporal pattern of the light sequence (e.g., the flash frequency or inter-stimulus interval) and the waveform (e.g., square, sinusoidal).
Relative or absolute size of the stimulus	Describe the size of the light stimulus, either absolute or relative (in relation to the visual field).

a Item reached consensus for being essential to report in any study regardless of experimental context.

b Luminance and radiance metrics (as opposed to illuminance and irradiance) are mainly relevant for emissive surfaces.

## Discussion

The ENLIGHT Checklist has been developed to provide guidance on the reporting of human laboratory studies deploying ocular light interventions. A modified four-step Delphi process was implemented, involving three questionnaire-based rounds and one round of face-to-face discussions. An initial list of 61 items was condensed into a final checklist of 25 items, 9 of which were determined by experts to be necessary to report regardless of the specific research question or context.

The ENLIGHT Checklist is a form-based fillable PDF and is available to download in Supplemental File S1. Authors are encouraged to fill in the checklist prior to the submission of a manuscript to ensure completeness in reporting. Researchers may also use the checklist to evaluate and organize the information critical to light studies when designing a study or preparing a grant application, although it should be noted that the checklist was designed to aid reporting and not the conduct of studies. In addition, the checklist is not intended to serve as a guide to evaluating design quality, only to facilitate complete reporting of relevant study and light characteristics and enhance reproducibility and external validity. The E&E document for completing the checklist is included in Supplemental File S2. The E&E document provides definitions and examples for items included in the checklist, along with additional resources and tools for calculating or understanding metrics referenced in the checklist. Many of the items included in the final version of the checklist do not require any special measurement equipment and can be documented and reported at no cost. Therefore, uptake of the ENLIGHT Checklist can be readily achieved. Ongoing feedback and suggestions can be submitted online as an “issue” on our GitHub repository (https://github.com/ENLIGHT-Project/ENLIGHT-Guidelines-Checklist), which will be used to inform potential revisions of the checklist and E&E document in the future. Although the ENLIGHT Checklist is intended to be used for human laboratory studies, modifications could be made to apply the checklist to other contexts, including field studies.

Achieving consensus within a community of researchers necessarily leads to compromises, not least due to heterogeneity and variability in backgrounds, interests, expertise, and training. A balance must be achieved between adequate and complete reporting, and the sometimes extensive resources required for particular measurements to be taken and metrics to be calculated. We, the ENLIGHT Steering Committee, believe the consensus reached shows an appropriate balance between these competing interests, given the current availability of resources and expertise. In our independent pilot testing phase, which involved an independent group of experts, the suitability of the ENLIGHT Checklist for use in the field has been confirmed.

We would like to note that one item which did not reach consensus but appeared multiple times in qualitative feedback from experts as an essential metric underpinning the reproducibility of studies. Spectral power distribution (in Section B.2. “*Light level characteristics*” of the checklist, Table 2) was rated by 73% of experts as essential to report in all contexts, falling just below the threshold for consensus (≥75%). Spectral measurements enable the calculation of many other metrics of interest, and therefore, are the simplest way to support reproducibility and comparison between studies. We acknowledge that the measurement of spectral distribution requires specialized equipment, which may not be available to all researchers, but nonetheless encourage authors to report this item whenever it is available.

Given the complex nature of measuring and reporting light metrics in human studies, the committee highlights that researchers and practitioners wishing to employ light as interventions must be appropriately trained in optical radiation metrology. During the consensus process, some experts highlighted that there is a lack of field-specific, accessible educational materials. The level of training necessary to perform some of the measurements poses a significant barrier to measuring and reporting specific metrics. The accompanying E&E document provides some tools and resources for understanding and calculating the items covered by the checklist. However, increasing accessible and appropriate education tools or materials will aid in increasing the ENLIGHT Checklist usage.

### Implementation plan

To ensure adoption of the ENLIGHT Checklist, we are following a multi-pronged approach:

• Engagement with journals to include ENLIGHT Checklist in author guidelines and requirements: We will seek adoption of the ENLIGHT Checklist by relevant journals in the field by contacting editors and journal offices upon publication of the ENLIGHT Checklist. We will additionally submit letters to the editor to specialized research journals that would benefit from incorporating the ENLIGHT checklist in research articles they receive.
• Engagement with funding organizations to implement the utilization of the ENLIGHT Checklist in grant applications and reporting: We are seeking adoption of the ENLIGHT Checklist by funding organizations. At the time of writing (October 2023), we already have agreement from one funder to endorse the ENLIGHT Checklist, and will pursue our efforts with additional national and international funding organizations. Due to the ENLIGHT Steering Committee's international make-up, we will be capable of reaching a wide range of funding organizations across the globe.
• Endorsement of ENLIGHT Checklist by scientific and professional associations and organizations: We are seeking approval and endorsement of the ENLIGHT Checklist by national, European and international scientific associations, asking them to promote the checklist upon publication, adding it prominently on their website, and adding their logo on the project website. In an early inquiry round prior to publication of this article, feedback from major scientific organizations in Europe, the US and Australia was generally positive, indicating that they will endorse, or consider endorsing, the checklist upon publication.
• Publication of an official international Technical Note by the International Commission on Illumination (CIE): The CIE is an international organization responsible for standards and quantities related to light and lighting. We are currently chairing a Division Reportership within CIE's Division 6 (Photobiology) to write a Technical Note detailing the ENLIGHT Checklist and E&E. The Technical Note will undergo an official approval and balloting process and be made available by the CIE. By elevating the checklist to a Technical Note, we are ensuring international visibility and ratification, thereby aiding uptake.
• Research community engagement and outreach: We will actively engage with relevant research communities through workshops, conferences, and online platforms. By presenting the checklist and its benefits in these settings, we hope to generate interest and discussion around its implementation.
• Project website inventorising checklists: Through our project website, we will maintain an active list of completed checklists for published papers. This will serve as a hub for information exchange and guidance and foster our collaborative approach.
• Social media and online campaigns: Utilizing social media platforms such as Twitter/X and LinkedIn, and online campaigns can create widespread awareness. Engaging with researchers, institutions, and the public through social media channels allows us to share updates, and relevant resources. Interactive Formats such as webinars can facilitate real-time discussions and address queries.
• Incorporating the ENLIGHT Checklist in academic training programs: Integration into academic curricula ensures that future researchers are well-acquainted with the ENLIGHT Checklist from the beginning of their careers. Collaborating with educational institutions, including the Steering Committee's own institutions, to incorporate the checklist into relevant courses, such as research methodology and scientific writing, creates a foundation of knowledge that aspiring researchers carry into their professional lives.

In our independent pilot testing phase, the ENLIGHT Checklist was positively appraised and considered to have the potential to improve reporting in the field by all respondents of the think-aloud sessions (n = 5), indicating the potential for the checklist to be impactful. Through the measures developed and detailed above, we are confident that we can ensure publicization, adoption, and use of the ENLIGHT Checklist.

### Outstanding questions

As the ENLIGHT Checklist is the first consensus checklist of its kind, there are a series of questions arising for future investigations:

**Standardization and adoption:** Which techniques can be used to encourage the adoption of the checklist? Individual researchers, institutions, funders, and journals all play a role in this, and it is likely that a concerted effort will yield success.

**Application in field and non-laboratory studies**: The ENLIGHT Checklist was developed with laboratory studies in mind. There is a growing literature on field or mixed studies examining the impact of light on humans in real-world scenarios, for which adapted versions of the checklist may be required.

**Assessment of impact on reporting quantity**: What methods or metrics will be used to assess the impact of the checklist on research reporting quality, and how will improvements be quantified?

**Continuous improvement:** How can feedback from the research community and checklist users be collected and used to iteratively improve both the checklist and the accompanying E&E document?

### Strengths and limitations

Our study exhibits several notable strengths. Firstly, the ENLIGHT Project adhered to a pre-registered protocol on the Open Science Framework, ensuring commitment to the initial research plan and bolstering the credibility of our findings. Our commitment to inclusivity was evident in our deliberate efforts to engage participants with diverse backgrounds, career stages, genders, and geographical locations. This approach significantly enriched the checklist by incorporating various perspectives and feedback, enhancing its quality and relevance. Furthermore, our selection of checklist items was grounded in a rigorous, evidence-based approach, drawing from a comprehensive array of references and expert input. Using a modified Delphi consensus process featuring multiple rounds of feedback and discussion with experts fortified the validity and reliability of both the checklist and guidelines, benefiting from the collective wisdom of a diverse group of experts. Our collaborative approach also garnered remarkably high participant satisfaction, reflecting the efficacy of our consensus-building process and the quality of the final checklist and guidelines E&E document. Our independent pilot testing phase, encompassing thinking-aloud sessions and written feedback from external experts, confirmed the usability and clarity of the ENLIGHT Checklist and E&E document. Lastly, our commitment to open science and reproducibility exemplifies our transparent practice of making all code, materials, and data accessible. These strengths collectively underscore the robustness and impact of our study.

Our study has a few limitations. Firstly, the clarity and usability of the ENLIGHT Checklist and E&E document have not been assessed by a broader group of external stakeholders. This evaluation is planned for future studies. However, during the pilot external/independent testing phase of this study, both the ENLIGHT Checklist and E&E document received positive feedback and were deemed to have the potential to enhance reporting in the field by all five respondents. Secondly, choosing a 75% inclusion threshold might seem arbitrary, but currently, there isn't a universally accepted standard method for setting consensus thresholds in reporting documents. While a threshold of 75% is not necessarily the standard for the modified Delphi process, it is in line with previous publications on consensus processes.^17^ Finally, even within a narrow field of research, reaching consensus among researchers with diverse backgrounds, interests, and expertise inevitably involves compromises due to this diversity. Balancing comprehensive reporting with the resources and expertise available to researchers is crucial. The ENLIGHT Steering Committee believes our consensus strikes an appropriate balance, considering current resources. The pilot independent testing phase confirms the suitability of the ENLIGHT Checklist for use in our field.

### Conclusion

The ENLIGHT Checklist is the first consensus-based checklist documenting and reporting light-based interventions for biomedical studies. The checklist and E&E document were derived through a systematic process involving in-depth interactions with experts in the field and future users of the checklist. Significant inter-individual differences exist in non-visual responses to light, and effects can be seen with even very low-level exposure. Therefore, minor differences in the delivery method, intensity, or spectral composition of light exposure may result in substantial variation in responses observed. In conclusion, the ENLIGHT Checklist represents a crucial step in improving the documentation of research on the physiological and biobehavioral effects of light, making this work more reproducible and fit for large-scale data synthesis.

## Contributors

*Conceptualization*: M.S., L.K., R.L., E.M., R.P.N.

*Data curation*: n/a.

*Formal Analysis*: n/a.

Funding *acquisition*: M.S., L.K., R.L., E.M., R.P.N.

*Investigation*: M.S., L.K., R.L., E.M., R.P.N.

*Methodology*: M.S., L.K., R.L., E.M., R.P.N.

*Project administration*: n/a.

*Resources*: n/a.

*Software*: n/a.

*Supervision*: n/a.

*Validation*: n/a.

*Visualization*: M.S., L.K., R.L., E.M., R.P.N.

*Writing—original draft*: M.S., L.K., R.L., E.M., R.P.N.

*Writing—review & editing*: M.S., L.K., R.L., E.M., R.P.N.

All authors have accessed the data. All authors read and approved the final version.

Across the four rounds of the Delphi process, invited experts contributed their time and expertise without remuneration and had the opportunity to opt into becoming a member of the ENLIGHT Consortium.

## Data sharing statement

See Methods, Code, materials and data availability.

## Declaration of interests

M.S.: None.

L.K.: None.

R.L.: None.

E.M.: None.

R.P.N.: None.

ENLIGHT Consortium: None.

## Appendix A Supplementary data

ENLIGHT_SupplementalMaterial

ENLIGHT_GroupAuthorship.docx

ENLIGHT_Checklist_Release

## References

[1] Vetter C., Pattison P.M., Houser K., Herf M., Phillips A.J.K., Wright K.P. (2022). A review of human physiological responses to light: implications for the development of integrative lighting solutions. Leukos.

[2] Blume C., Garbazza C., Spitschan M. (2019). Effects of light on human circadian rhythms, sleep and mood. Somnologie.

[3] Duffy J.F., Czeisler C.A. (2009). Effect of light on human circadian physiology. Sleep Med Clin.

[4] Lok R., Smolders K.C.H.J., Beersma D.G.M., de Kort Y.A.W. (2018). Light, alertness, and alerting effects of white light: a literature overview. J Biol Rhythms.

[5] Cajochen C., Zeitzer J.M., Czeisler C.A., Dijk D.J. (2000). Dose-response relationship for light intensity and ocular and electroencephalographic correlates of human alertness. Behav Brain Res.

[6] Vandewalle G., Maquet P., Dijk D.-J. (2009). Light as a modulator of cognitive brain function. Trends Cogn Sci.

[7] Chellappa S.L. (2021). Individual differences in light sensitivity affect sleep and circadian rhythms. Sleep.

[8] Spitschan M., Santhi N. (2022). Individual differences and diversity in human physiological responses to light. eBioMedicine.

[9] Phillips A.J.K., Vidafar P., Burns A.C., McGlashan E.M., Anderson C., Rajaratnam S.M.W. (2019). High sensitivity and interindividual variability in the response of the human circadian system to evening light. Proc Natl Acad Sci U S A.

[10] Brown T.M. (2020). Melanopic illuminance defines the magnitude of human circadian light responses under a wide range of conditions. J Pineal Res.

[11] Logullo P., MacCarthy A., Kirtley S., Collins G.S. (2020). Reporting guideline checklists are not quality evaluation forms: they are guidance for writing. Health Sci Rep.

[12] Veitch J.A., Knoop M. (2020).

[13] Spitschan M., Stefani O., Blattner P., Gronfier C., Lockley S., Lucas R. (2019). How to report light exposure in human chronobiology and sleep research experiments. Clocks Sleep.

[14] Remé C., Menozzi M., Krueger H. (1991). Standards for specifying parameters of bright light stimulation for therapeutic applications: a proposal. Bull Soc Light Treatment Biol Rhythm.

[15] Knoop M., Broszio K., Diakite A., Liedtke C., Niedling M., Rothert I. (2019). Methods to describe and measure lighting conditions in experiments on non-image-forming aspects. Leukos.

[16] Okoli C., Pawlowski S.D. (2004). The Delphi method as a research tool: an example, design considerations and applications. Inf Manag.

[17] Diamond I.R., Grant R.C., Feldman B.M., Pencharz P.B., Ling S.C., Moore A.M. (2014). Defining consensus: a systematic review recommends methodologic criteria for reporting of Delphi studies. J Clin Epidemiol.

