# Supplementary material for: ENLIGHT: A consensus checklist for reporting laboratory-based studies on the non-visual effects of light in humans
Source: eBioMedicine. 2023 Dec 2;98:104889. doi: 10.1016/j.ebiom.2023.104889 (PMC10704221; doi:10.1016/j.ebiom.2023.104889)
Supplement: ENLIGHT_SupplementalMaterial [file mmc1.pdf]

ENLIGHT: A consensus checklist for reporting laboratory-based studies on the non-visual effects of light in humans

Supplemental Material

Manuel Spitschan [0000-0002-8572-9268][1, 2, 3, 4, +, *]

Laura Kervezee [0000-0002-6062-9164][5, +, *]

Renske Lok [0000-0003-1684-5625][6, +, *]

Elise McGlashan [0000-0002-3864-7198][7, 8, +, *]

Raymond P. Najjar [0000-0002-3770-2300][9, 10, 11, 12, +, *]

for the ENLIGHT Consortium[¶]

[1] TUM School of Medicine & Health, Department of Health and Sport Sciences, Technical University of Munich, Munich, Germany

[2] TUM Institute for Advanced Study (TUM-IAS), Technical University of Munich, Garching, Germany

[3] Max Planck Institute for Biological Cybernetics, Max Planck Research Group Translational Sensory & Circadian Neuroscience, Tübingen, Germany

[4] TUMCREATE, Singapore, Singapore

[5] Laboratory for Neurophysiology, Department of Cellular and Chemical Biology, Leiden University Medical Center, Leiden, Netherlands

[6] Department of Psychiatry and Behavioral Sciences, Stanford University, Stanford, USA

[7] Melbourne School of Psychological Sciences, The University of Melbourne, Parkville, Victoria, Australia

[8] School of Psychological Science and Turner Institute for Brain and Mental Health, Monash University, Melbourne, Australia

[9] Department of Ophthalmology and Department of Biomedical Engineering, National University of Singapore, Singapore, Singapore

[10] Center for Innovation & Precision Eye Health, National University of Singapore, Singapore, Singapore

[11] Singapore Eye Research Institute, Singapore, Singapore

[12] Ophthalmology and Visual Sciences Academic Clinical Programme, Duke-NUS Medical School, Singapore, Singapore

+ Equal contribution

* To whom correspondence may be addressed: manuel.spitschan@tum.de; L.Kervezee@lumc.nl; rlok@stanford.edu; elise.mcglashan@monash.edu; rpnajjar@nus.edu.sg

¶ The full list of ENLIGHT Consortium members can be found in **Supplemental Table S2** of this manuscript.

Supplemental Material

Supplemental Figure S1: Ratings of inclusion and exclusion of items in the initial draft checklist (Round 2).

Supplemental Figure S2: Format preference of items in the initial draft checklist (Round 2).

Supplemental Figure S3: Percentage of votes for essential reporting of items (Round 4).

Supplemental Table S1: Summary of reported light metrics in 19 studies described in Brown (2020).

Supplemental Table S2: ENLIGHT Consortium Members.

Supplemental Table S3: Common clusters of feedback raised in face-to-face discussion sessions in Round 3.

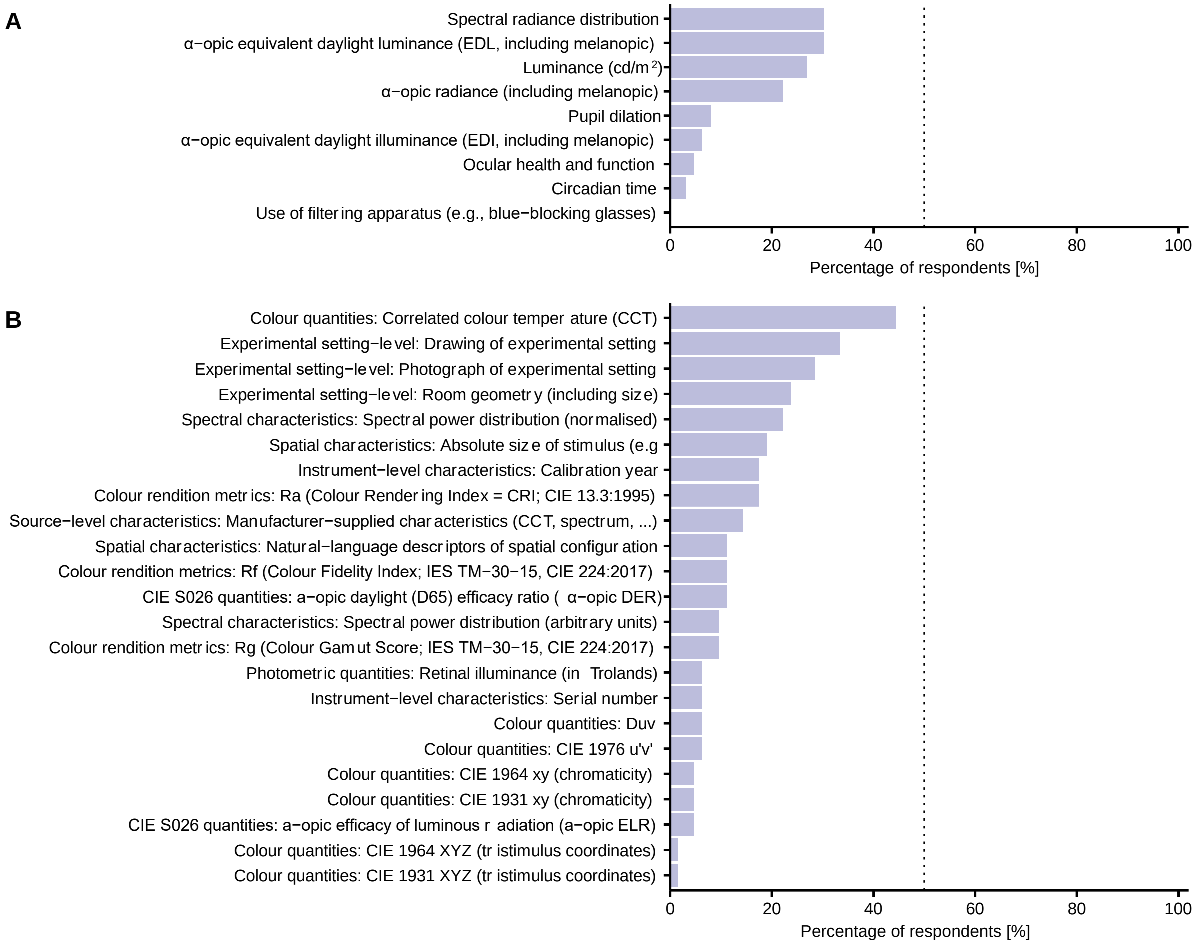

Supplemental Figure S1: Ratings of inclusion and exclusion of items in the initial draft checklist in Round 2 (n = 63 participants). (A) Percentage of participants that rated a post-hoc-included item as "should not have been included". (B) Percentage of participants that rated an excluded item as "should have been included".

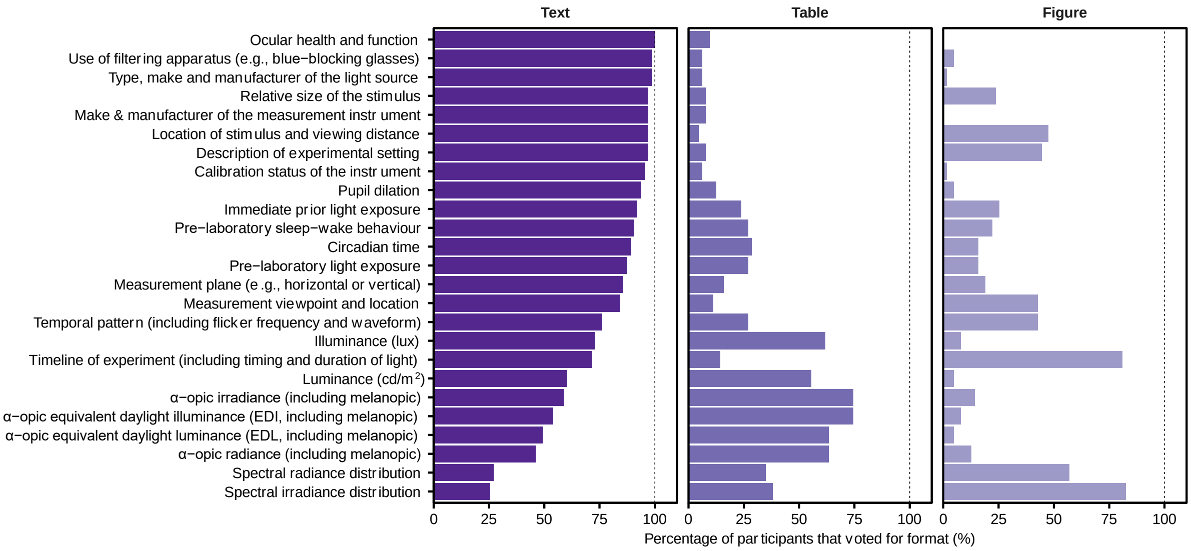

Supplemental Figure S2: Format preference of items in the initial draft checklist as rated by participants (n = 63 participants) in Round 2.

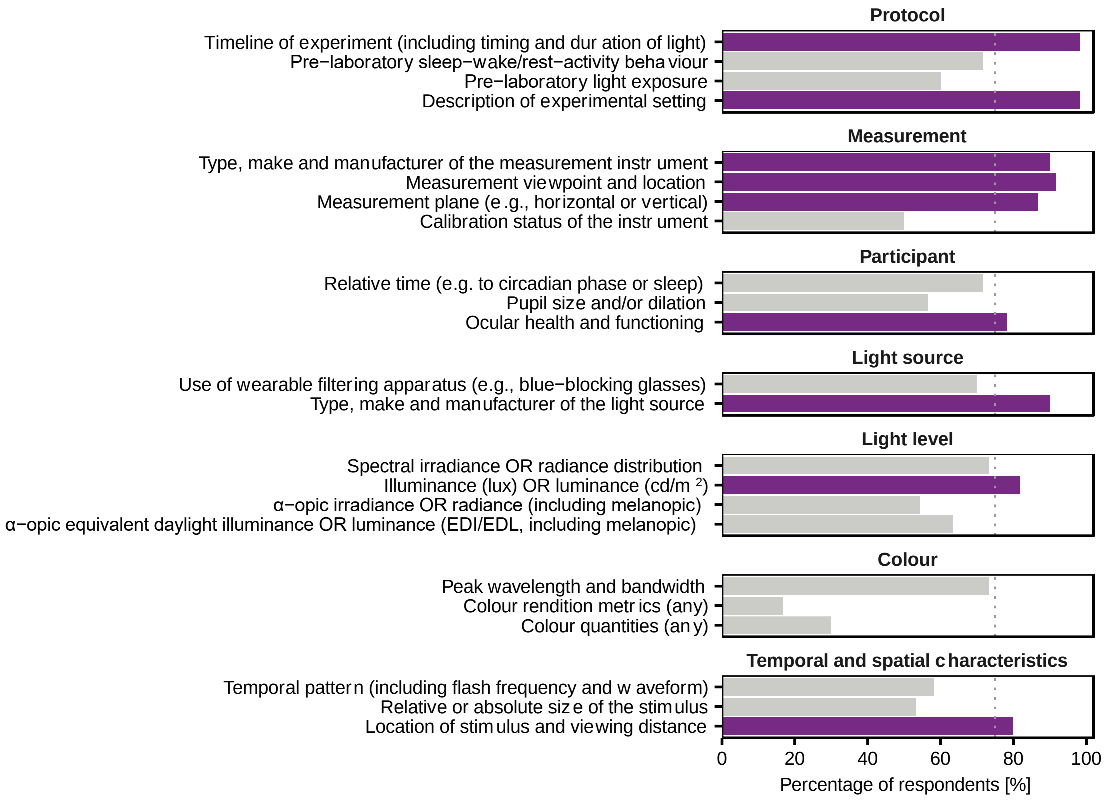

Supplemental Figure S3: Percentage of votes for essential reporting of items regardless of experimental context (n = 60 participants in Round 4). Dark purple bars: items that reached consensus on being essential (i.e., were considered essential by more than 75% of respondents). Light gray bars: items that were voted by at most 75% of respondents as essential and therefore did not reach consensus on being essential to report regardless of context.

Supplemental Table S1. Summary of reported light metrics of 19 laboratory studies (referenced in Brown, 2020[#]) investigating the effects of light on melatonin suppression and/or circadian phase resetting in response to at least two spectrally distinct light sources delivered at multiple intensities. Two members of the ENLIGHT Steering Committee summarized these articles for reporting the items in the table. Results were then cross-referenced between members.

Category	Output measure	No. of studies reporting (out of 19)
Absolute spectrally unweighted intensities	Irradiance (energy units)	10
	Irradiance (photon units)	11
	Radiance (energy units)	0
	Radiance (photon units)	0
Light intensity	Illuminance (lux)	14
	Luminance (cd/m^2)	1
	Luminous flux (lumens)	0
	Any other luminance-like quantity	1
	Melanopic quantity	8
	Chromaticity (x,y, chromaticity coordinates)	0
	Correlated Colour Temperature	8
	Type of spectrum used	19 (monochromatic: 10)
Narrowband spectra	Peak wavelength given	10
	Bandwidth given	10
Spectral power distribution	Absolute spectrum given (tabulated)	2
	Relative spectrum given (tabulated)	0
	Absolute spectrum given (graph)	3
	Relative spectrum given (graph)	9

[#]Brown TM. Melanopic illuminance defines the magnitude of human circadian light responses under a wide range of conditions. *J Pineal Res*. 2020;69(1):e12655.

Supplemental Table S2: ENLIGHT Consortium members.

Last name	First name	ORCID
Allen	Annette E.	0000-0003-0214-7076
Andersen	Marilyne	0000-0001-8813-1184
Bará	Salvador	0000-0003-1274-8043
Blattner	Peter	0000-0003-0681-2096
Blume	Christine	0000-0003-2328-9612
Boivin	Diane B.	0000-0002-3896-2710
Bonmatí-Carrión	María-Ángeles	0000-0002-9050-6448
Broszio	Kai	0000-0002-8269-8654
Brown	Timothy M.	0000-0002-5625-4750
Chellappa	Sarah Laxhmi	0000-0002-6190-464X
Duffy	Jeanne F.	0000-0003-4177-4179
Eto	Taisuke	0000-0002-0347-7408
Flynn-Evans	Erin	-
Fotios	Steve	0000-0002-2410-7641
Gabel	Virginie	0000-0002-9792-1468
Garbazza	Corrado	0000-0002-8606-2944
Glickman	Gena	0000-0001-7064-7238
Gordijn	Marijke C.	0000-0001-9521-8085
Hanifin	John P.	-
Hartstein	Lauren	0000-0002-3643-8894
Herf	Michael	-
Higuchi	Shigekazu	0000-0001-7131-0792
Hilditch	Cassie J.	0000-0001-8797-4390
Houser	Kevin W.	0000-0001-6097-1560
Hurlbert	Anya	0000-0002-9879-5758
LeBourgeois	Monique K.	-
Lockley	Steven	0000-0001-5209-2881
Lucas	Robert	0000-0002-1088-8029

– Supplemental table S1 continues on next page –

– Supplemental table S1 continued from previous page –

Moreno	Claudia R. C.	0000-0003-1839-9673
Münch	Mirjam	0000-0003-2087-9916
Mure	Ludovic S.	0000-0002-2485-1032
Peirson	Stuart	0000-0003-3653-834X
Rahman	Shadab	0000-0002-8729-0500
Revell	Victoria L.	0000-0002-8809-4587
Rodriguez	Roberto G.	0000-0001-7888-2152
Roecklein	Kathryn	0000-0001-5206-8283
Rukmini	A. V.	0000-0002-1300-7442
Sammarco	John	0000-0001-5843-634X
Santhi	Nayantara	-
Schlangen	Luc J. M.	0000-0002-8424-7240
Schöllhorn	Isabel	0000-0001-9067-1614
Sharkey	Katherine M.	0000-0003-1743-0704
Skene	Debra J.	0000-0001-8202-6180
Sletten	Tracey L.	0000-0002-0005-7838
Smolders	Karin C. H. J.	0000-0001-5838-8501
Stefani	Oliver	0000-0003-0199-6500
Stone	Julia E.	0000-0001-7706-7471
Teikari	Petteri	0000-0003-1095-4185
Terman	Michael	0000-0002-8871-3018
Tran Quoc	Khanh	-
Tsubota	Kazuo	0000-0002-8874-7111
Udovicic	Ljiljana	0000-0002-6035-8309
Vandewalle	Gilles	0000-0003-2483-2752
Veitch	Jennifer A.	0000-0003-3183-4537
Vetter	Céline	0000-0002-3752-1067
Wu	Lisa M.	0000-0003-2241-9905

– Supplemental table S1 continues on next page –

– *Supplemental table S1 continued from previous page* –

Zauner	Johannes	0000-0003-2171-4566
Zeitzer	Jamie	0000-0001-6174-5282

Supplemental Table S3. Summary of the most common feedback, suggestions, and actions the steering committee took following the face-to-face discussions in Round 3.

	**Feedback from ENLIGHT Consortium**	**Actions taken by the ENLIGHT Steering Committee**
**Objective 1:** To clarify open questions and concerns from participants	The checklist should be concise.	To ensure conciseness, the following items were removed:<br>- Items that describe similar or alternative metrics.<br>- Preferred format (table, text, figure).
	Avoid the checklist being considered prescriptive or used to evaluate study quality.	It was clarified in the Explanation and Elaboration (E&E) document and manuscript that the checklist does not intend to evaluate study quality. The checklist is meant to provide a space for authors to indicate whether and where metrics are described.
	Clarify whether NA means "not applicable" or "not available".	Both "not applicable", and "not available" were added to the checklist, as the committee appreciated that both responses may be relevant across items, and it is important to provide the authors the opportunity to indicate either.
	Some light metrics may not be reportable by labs with limited resources.	As highlighted in the E&E document, most items included on the checklist can be measured and reported with minimal resources to ensure the checklist is as accessible as possible.
	The rationale regarding items included post-hoc following Round 1 should be clarified to avoid subjectivity bias.	Following Round 1, none of the items included in the checklist were deemed by participants to be "unimportant" or "should not be included". However, some aspects of lighting where *no individual items* reached the threshold for *definite inclusion (i.e., 75% consensus)*. Additional items that fell minimally below the threshold were added to the checklist, to ensure all aspects of light were covered. In Round 2, we asked participants to indicate whether they felt that post hoc included items should *not have been included*. For each item, less than 31% of experts rated them as "should not have been included", and therefore all were retained.

**Objective 2:** To discuss the scope of the E&E document accompanying the checklist	The E&E document should also highlight why taking the light measurements specified in the checklist is important.	The description of why metrics or items are important is beyond the scope of the E&E document and checklist. This study aims to reach consensus among experts on items to be reported in manuscript to enhance reproducibility and external validity of studies in the research field.
	The E&E document should mention that the objectives of the checklist are to enhance reproducibility and external validity of findings.	The objectives of the E&E document were revised to reaffirm the rationale and intention of the checklist and E&E.
	The E&E document should address that some metrics are redundant and can be converted from other metrics.	Resources for converting metrics are provided in the E&E document, and this has been highlighted in the manuscript.
	The E&E document could include examples to indicate what quantity is important to report in what experiment, and which are critical/strongly recommended or optional.	The consensus around which items should be reported regardless of context (i.e., to remove the 'not applicable' checkbox for those items") was achieved in Round 4.<br><br>In addition, we have also clarified in the checklist and E&E document the contexts in which the use of irradiance/illuminance versus radiance/luminance metrics may apply.
	The E&E document should include sufficient background information so that it is clear to researchers who are new to the field.	In the E&E document, we have explained the concepts included in the checklist as clearly and concisely as possible, and provided additional links to materials that may be useful.
**Objective 3:** To discuss how to maximize impact and facilitate the adoption of the ENLIGHT Checklist and E&E document.	Several participants across sessions agreed that the checklist, E&E document, and any publications resulting from this work should be open access to ensure widespread availability. They suggested that endorsement by organizations, research groups, and conferences could increase visibility, and that social media should be used to promote it. In addition, they emphasized the importance of gaining the support of journal editors, as a crossover between communities tends to be limited, and a snowball effect could be achieved by following the manuscript with multiple editorials.	
