# Supplementary material for: ENLIGHT: A consensus checklist for reporting laboratory-based studies on the non-visual effects of light in humans
Source: eBioMedicine. 2023 Dec 2;98:104889. doi: 10.1016/j.ebiom.2023.104889 (PMC10704221; doi:10.1016/j.ebiom.2023.104889)
Supplement: ENLIGHT_GroupAuthorship.docx [file mmc2.docx]

| **First name** | **Last name** |  |
| --- | --- | --- |
| Annette E. | Allen |  |
| Marilyne | Andersen |  |
| Salvador | Bará |  |
| Peter | Blattner |  |
| Christine | Blume |  |
| Diane B. | Boivin |  |
| María-Ángeles | Bonmatí-Carrión |  |
| Kai | Broszio |  |
| Timothy M. | Brown |  |
| Sarah Laxhmi | Chellappa |  |
| Jeanne F. | Duffy |  |
| Taisuke | Eto |  |
| Erin | Flynn-Evans |  |
| Steve | Fotios |  |
| Virginie | Gabel |  |
| Corrado | Garbazza |  |
| Gena | Glickman |  |
| Marijke C. | Gordijn |  |
| John P. | Hanifin |  |
| Lauren | Hartstein |  |
| Michael | Herf |  |
| Shigekazu | Higuchi |  |
| Cassie J. | Hilditch |  |
| Kevin W. | Houser |  |
| Anya | Hurlbert |  |
| Monique K. | LeBourgeois |  |
| Steven | Lockley |  |
| Robert | Lucas |  |
| Claudia R. C. | Moreno |  |
| Mirjam | Münch |  |
| Ludovic S. | Mure |  |
| Stuart | Peirson |  |
| Shadab | Rahman |  |
| Victoria L. | Revell |  |
| Roberto G. | Rodriguez |  |
| Kathryn | Roecklein |  |
| A. V. | Rukmini |  |
| John | Sammarco |  |
| Nayantara | Santhi |  |
| Luc J. M. | Schlangen |  |
| Isabel | Schöllhorn |  |
| Katherine M. | Sharkey |  |
| Debra J. | Skene |  |
| Tracey L. | Sletten |  |
| Karin C. H. J. | Smolders |  |
| Oliver | Stefani |  |
| Julia E. | Stone |  |
| Petteri | Teikari |  |
| Michael | Terman |  |
| Khanh | Tran Quoc |  |
| Kazuo | Tsubota |  |
| Ljiljana | Udovicic |  |
| Gilles | Vandewalle |  |
| Jennifer A. | Veitch |  |
| Céline | Vetter |  |
| Lisa M. | Wu |  |
| Johannes | Zauner |  |
| Jamie | Zeitzer |  |
